# Uterine PEComa: appraisal of a controversial and increasingly reported mesenchymal neoplasm

**DOI:** 10.1186/1477-7800-5-7

**Published:** 2008-03-06

**Authors:** Oluwole Fadare

**Affiliations:** 1 Department of Pathology, Wilford Hall Medical Center, Lackland Air Force Base, San Antonio, TX 78236, USA; 2 Department of Pathology, University of Texas Health Science Center at San Antonio, San Antonio, TX 78229, USA

## Abstract

In recent years, a group of tumors that have been designated "perivascular epithelioid cell tumors" (PEComa) have been reported with increasing frequency from a wide variety of anatomic locations. The uterus and retroperitoneum appear to be the most frequent sites of origin for these lesions. PEComas belong to an identically named family of tumors comprised of conventional angiomyolipomas, clear cell sugar tumors, lymphangiomyomatosis and clear cell myomelanocytic tumor of the falciform ligament/ligament teres, and are also known as PEComa-NOS. This article is a primer for clinicians on the most salient clinicopathologic features of uterine PEComas, as most of the debate and discussion have taken place in the pathologic literature. The author appraises in detail the current state of knowledge on PEComas of the uterus based on a review of published data on the 44 previously reported cases, and comments on areas of controversy. The latter are centered predominantly on the significant morphologic and immunophenotypic overlap that exists between uterine PEComa and some smooth muscle tumors of the uterus. The clinicopathologic features of cases reported as epithelioid smooth muscle tumors and cases reported as uterine PEComas are compared and contrasted, and a practical approach to their reporting is proposed.

## Background

In 1994, Bonetti et al [1] proposed the concept of a family of tumors comprised of angiomyolipoma, clear cell sugar tumors and lymphagioleiomyomatosis. The proposal was based on observations that these 3 lesions shared a morphologically and immunophenotypically distinctive cell type which the authors had previously designated "perivascular epithelioid cell" [2,3]. Cells that are probably synonymous with these perivascular epithelioid cells (PECs) were first noted in renal angiomyolipomas by Apitz in 1943 [4]. These cells lack a normal anatomic homologue, have spindle to epithelioid shapes with clear to eosinophilic cytoplasm, display a predilection for perivascular arrangements, and display immunoreactivity for melanocytic markers such as HMB-45, micropthalmia transcription factor (miTF), Melan A, Mart-1, HMSA-1 and to a lesser extent, muscular markers such as actin and desmin [5-15]. PECs are envisioned by the Bonetti group as possessing a phenotypic plasticity wherein the cells may assume a spindled appearance and be more likely to be positive for muscular markers, an epithelioid appearance associated with a higher frequency of immunoreactivity for melanocytic markers, or various phenotypic modulations in between [14,15]. Over the subsequent one and a half decades, an increasing number of neoplasms putatively composed of PECs have been reported from a wide variety of anatomic locations under a similarly wide variety of appellations. The term PEComa was introduced in 1996 by Zamboni et al to describe one such case arising in the pancreas [16]. Perivascular epithelioid cell tumors received formal recognition in 2 monographs published under the auspices of the World Health Organization (WHO) in 2002 and 2003. [10,17]. In the WHO classification of soft tissue neoplasms [10], PEComa was defined as "mesenchymal tumors composed of histologically and immunohistochemically distinctive perivascular epithelioid cells", including angiomyolipoma, clear cell sugar tumors, lymphagioleiomyomatosis, clear cell myomelanocytic tumor of the falciform ligament/ligament teres and other unusual clear cell tumors at various locations [10]. Clear cell myomelanocytic tumor of the falciform ligament/ligament teres [18] is presently not considered sufficiently distinctive and is now generally included in the latter group of "unusual clear cell tumors" at various locations. The designation PEComa-NOS (perivascular epithelioid cell tumors-not otherwise specified) has been applied to these "unusual clear cell tumors" to avoid applying the same designation – PEComa – to the family of lesions as well as a constituent subset [5-8]. However, the unqualified "PEComa" designation is recognized in the WHO monographs [10,17] and will be used henceforth in this article to describe the aforementioned constituent subset. PEComas display an overwhelming female preponderance and appear to be anatomically ubiquitous [5,11-15]. However, the uterus and retroperitoneum have emerged as the 2 most frequently reported sites of origin for these neoplasms [5,7,8,12]. Notably, most of the uterine cases were described within the past decade [8]. The first uterine PEComa was described in 1992 [19], and 43 additional cases have subsequently been reported in the English literature [5,13,20-41]. In this article, the author evaluates the current state of knowledge on PEComas of the uterus, with an emphasis on controversial areas and their unclear relationship with uterine smooth muscle neoplasia.

## Uterine PEComas: clinical features

Clinical features of the 44 previously reported cases of uterine PEComa are summarized in Table 1. These cases include tumors reported as PEComa, perivascular epithelioid cell tumor, abdominopelvic sarcoma, hyalinized uterine mesenchymal neoplasms with HMB-45-positive epithelioid cells, epithelioid angiomyolipoma [5,13,19-41], and excludes cases reported as conventional angiomyolipoma, lipoleiomyomas and lymphangioleiomyomatosis of the uterus [42-49]. One case that was originally reported as lymphangioleiomyomatosis [50], but which was subsequently included in a series of PEComas [33], is included. Only cases reported prior to January 31, 2008 in the English literature are included.

**Table 1:** Clinical features of the 44 reported cases of uterine PEComa***

**REFERENCE**	**Year**	**Age (yrs)**	**TSC**	**Location**	**Management**	**Outcome**
Hornick & Fletcher (39)	2008	34	NS	Corpus	NS	NERM at 33 months
Armah & Parwani (40)	2008	59	No	Corpus	Hysterectomy/BSO performed 7-years previously for presumed leiomyosarcoma; Renal and pulmonary metastases at current presentation; all resected	NERM at 15 months
Liang et al (41)	2008	59	Yes	Corpus	Hysterectomy/BSO/Pelvic and paraaortic Lymphadenenctomy, omentectomy, appendectomy, peritoneal biopsies; hormonal therapy	Extension into cervix at presentation; NERM at 10 months
Gan et al (20)	2007	40	NS	Corpus	Hysterectomy	NERM at 16 months
		33	NS	Corpus	Hysterectomy	NERM at 32 months
		44	NS	Corpus	Hysterectomy	NERM at 52 months
Rammeh Rommani et al (21)	2006	35	No	Corpus	Excision	NERM at 4 months
Azad et al (22)	2006	25	No	Cervix	Radical hysterectomy, Lymphadenectomy	Unavailable or Recent
Folpe et al (13)	2005	59	No	Corpus	Adjuvant Chemotherapy	Liver and Lung metastases at 30 months. Alive with metastases 30 months
		56	No	Corpus	Adjuvant Chemotherapy and radiotherapy	Lung and bone metastases at 11 months; Alive with metastases 11 months
		36	No	Corpus	Hysterectomy, adjuvant chemotherapy	Lung metastases at 12 months, liver metastases 36 months; Death at 39 months
		42	No	Corpus	No additional surgery or adjuvant therapy	NERM at 11 months
		28	No	Cervix	Hysterectomy and Lymphadenectomy	NERM at 36 months
		48	No	Cervix	Adjuvant Radiotherapy	NERM at 21 months
Jeon et al (23)	2005	9	No	Corpus	Neoadjuvant chemotherapy, Hysterectomy with Lymphadenectomy, Adjuvant Chemotherapy/Radiotherapy	Lymph node metastases at presentation, NERM at 18 months
Bernado Vega et al (27)	2005	34	No	Corpus	Hysterectomy/BSO	Unavailable or Recent
Bosincu et al (24)	2005	59	No	Corpus	Hysterectomy/BSO, omentectomy, abdominal exploration	Pelvic recurrence at 6 months; Death at 12 months
		48	No	Corpus	GnRH analogues, then Hysterectomy	NERM at 36 months
Fukunaga (25)	2005	40	No	Corpus	Hysterectomy/BSO, omentectomy, Adjuvant chemotherapy and radiation.	Ovarian and Omental metastases at presentation, Death at 16 months
		30	No	Corpus	Hysterectomy	NERM at 36 months
		36	No	Corpus	Hysterectomy	NERM at 6 months
Fukunaga (25,26)	2004	32	No	Corpus	Excision	NERM at 12 months
Gao et al (28)	2004	60	No	Corpus	Hysterectomy/BSO, staging	Unavailable or Recent
Darai et al (29)	2004	18	No	Corpus	Excision	Pelvic recurrence and lymph node metastases, 6 months, NERM subsequent 24 months
Fadare et al (5)	2004	41	Yes	Cervix	Hysterectomy/BSO	NERM at 35 months
Dimmler et al (32)	2003	61	NS	Corpus	Hysterectomy	Lung metastases at 84 months
Greene et al (30)	2003	79	NS	Corpus	Hysterectomy/BSO	Pelvic and abdominal recurrence at 24 months; Death at 24+ months
Park et al (31)	2003	32	No	Corpus	Hysterectomy/RSO	Right broad ligament/mesovarian extension at presentation; NERM at 18 months
Vang & Kempson (33)	2002	40	No	Corpus	Excision	Unavailable or Recent
		54	No	Corpus	Hysterectomy/BSO	Unavailable or Recent
		56	No	Corpus	Hysterectomy/BSO	Unavailable or Recent
		75	No	Corpus	Hysterectomy/BSO, Adjuvant Radiotherapy	NERM at 31.2 months
		47	No	Corpus	Hysterectomy/BSO	NERM at 1.5 months
		49	Yes	Corpus	Hysterectomy/BSO	NERM at 54 months
		55	No	Corpus	Hysterectomy/BSO	NERM at 2 months
		58	No	Corpus	Hysterectomy/BSO	Unavailable or Recent
Bonnetti et al (34)	2001	19	No	Corpus	Hysterectomy/BSO/pelvic and inguinal lymphadenectomy, Adjuvant chemotherapy and radiotherapy	Metastases to pelvic/inguinal nodes and vaginal extension at presentation. Local recurrence, bone and lung metastases 18 months; no further follow-up
		41	Yes	Corpus	Hysterectomy/BSO	Ovarian metastases at presentation, NERM at 6 months
Michal & Zamecnik* (35)	2000	58	No	Corpus	Hysterectomy	NERM at 48 months
		48	No	Corpus	Hysterectomy	NERM at 48 months
		46	No	Corpus	Hysterectomy	NERM at 12 months
		46	No	Corpus	Hysterectomy	NERM at 12 months
Ruco et al** (38)	1998	56	No	Corpus	NS	Ovary and ileum metastases at presentation
Pea et al (19)	1996	57	No	Corpus	NS	NERM at 24 months

TSC tuberous sclerosis complex; BSO bilateral salpingo-oophorectomy; NERM no evidence of tumor recurrence or metastases. *these authors [35] published a subsequent opinion in which they indicated that their cases are better designated epithelioid smooth muscle neoplasia [36].

**Presumes the same case is being described in references 37 and 38. ***Includes cases published in the English literature before 01/31/08 only, and excludes the case reported in reference 53 due to lack of data.

Forty and four of these 44 cases arose in the uterine corpus and uterine cervix respectively. The patients ranged in age from 9 to 79 (mean 45). The presentations were wide and varied, and included abnormal vaginal bleeding, abdominopelvic pain, uterine rupture, and hemoperitoneum; the tumors were incidental discoveries in several cases [5,13,19-41]. The radiological appearances have been similarly varied and appear to be largely dependent on the biologic characteristics of the underlying tumors. They may be small and homogeneous, simulating a benign smooth muscle neoplasm or they may be large, lobulated and heterogeneous masses [51]. Overall, neither the clinical presentation nor radiologic appearance of uterine PEComas is sufficiently distinctive to allow the diagnosis to be suggested preoperatively.

Preliminary data is suggestive of a possible association between uterine PEComa and the tuberous sclerosis complex (TSC). TSC is an autosomal dominant syndrome, one of the phacomatoses, that may be characterized by a wide variety of neoplastic manifestations, including renal angiomyolipomas, lymphangioleiomyomatosis, cardiac rhabdomyomas, subependymal giant cell astrocytoma as well as several others [52]. Four (9.1%) of the 44 patients with uterine PEComas also had the TSC [5,33,34]. Although this rate of association is probably inflated due to the selection bias associated with the reported cases, it is still notably higher than any other neoplastic process of the uterus, and suggests that patients with uterine PEComa be briefly evaluated for the stigmata of this complex. Lymphangioleiomyomatosis, another characteristic manifestation of TSC, was identified in the lymph nodes of 3 (6.8%) of the 44 patients [33,41], 2 of whom had TSC. The vast majority of the 44 patients received surgical management that included at least a hysterectomy.

The reported uterine PEComas have displayed a spectrum of biologic behaviors, and any discussions about the "prognosis" of PEComas as a single neoplasm is fraught with the same fallacies of rendering a blanket statement about the prognosis of a heterogeneous group such as uterine smooth muscle tumors, for example. Follow-up information and/or manifest malignancy at presentation were available in 37 (84%) of the 44 patients. In a previous report [8], a subset of these cases (corpus only) was classified into 2 groups based on patient outcome. The first group was designated "malignant" and was comprised of cases associated with patient death of disease and/or extrauterine extension at presentation. The second group was designated "non-malignant" and was comprised of cases in which neither of the aforementioned features was present [8]. After updating that paradigm with cases reported since the report, 15 (44%) of the 34 corpus cases (with follow-up and/or manifest malignancy at presentation) may be classified as malignant and the remaining 19 (56%) as non-malignant. If the 3 cases primary in the cervix and with follow-up information are included, there would be 16 (43%) of 37 cases in the malignant group and 21 (57%) of 37 cases in the non-malignant group. There is no statistically significant difference in patient age between the 2 groups. The above analysis excludes the uterine case included in the series of Pan et al due to lack of specific data [53].

## Uterine PEComas: pathologic features

**Figure 1:**
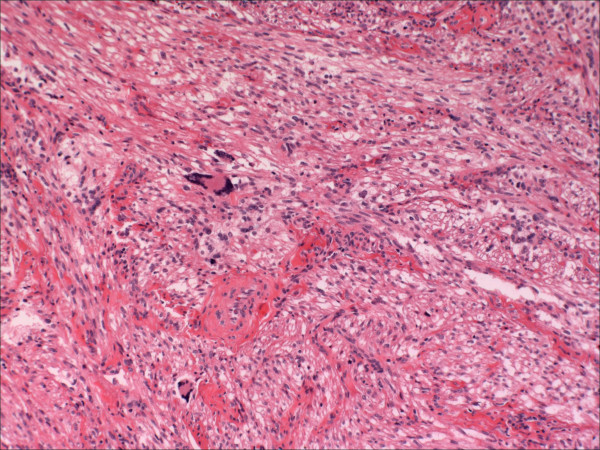
A perivascular epithelioid cell tumor of the uterine cervix that pursued a benign clinical course. Note the network of capillaries and the multinucleated giant cells. (from ref 5)

The uterine tumors reported as PEComas have been fundamentally characterized by a diffuse, nested and/or fascicular proliferation of spindled and epithelioid cells that display clear to eosinophilic cytoplasms, and which display immunoreactivity for melanocytic markers [5,13,19-41]. A given lesion may be dominated by spindled cells or epithelioid cells, but these cells are often admixed [8,13]. Some cases are comprised of cells with bland nuclei whereas others display frank anaplasia; most fall somewhere within this spectrum. Multinucleated giant cells, which appear to be degenerative in nature may also be found [5,8,13,41] (Figure 1). The constituent cells may display a perivascular distribution, but this feature is often inconspicuous in the uterine cases [8]. PEComas typically display a prominent network of small capillaries reminiscent of renal clear cell carcinoma or myxoid liposarcoma (Figure 1). The tumors may be circumscribed with no or minor areas of peripheral infiltration, or display a prominent "tongue-like" infiltration into the myometrium [8,33]. Two cases [5,41] were associated with clusters of epithelioid cells outside of the main tumoral mass, including the ovary and small bowel in one case [5]. This phenomenon was designated "PEComatosis" in one 2004 report [5]. PEComas may display stromal hyalinization that can be so diffuse as to obfuscate their underlying features [19,25,33,35,39,41]. In addition to HMB-45, PEComas also display immunoreactivity, albeit in lesser proportions, to other melanocytic markers such as micropthalmia transcription factor, Melan A, Mart-1, and HMSA-1. Greater than 70% are positive for smooth muscle actin (SMA), and almost half are positive for desmin [8]. The full immunophenotype of uterine PEComas is outlined in Table 2.

**Table 2:** The immunophenotypic profile of the reported uterine PEComas**

**Antibody**	**Percentage positive**
HMB-45	100
Smooth muscle actin	73
Vimentin	56
Desmin	49
Muscle-specific actin	36
CD10	25
Melan-A	24
CD117	9
CD34	5
Chromogranin	0
S100	3
Keratin	3
Epithelial Membrane Antigen	0
Inhibin	0

**Data from Fadare (8), and only includes markers for which at least 10 cases have been tested. Any extent or intensity of staining considered positive

**Figure 2:**
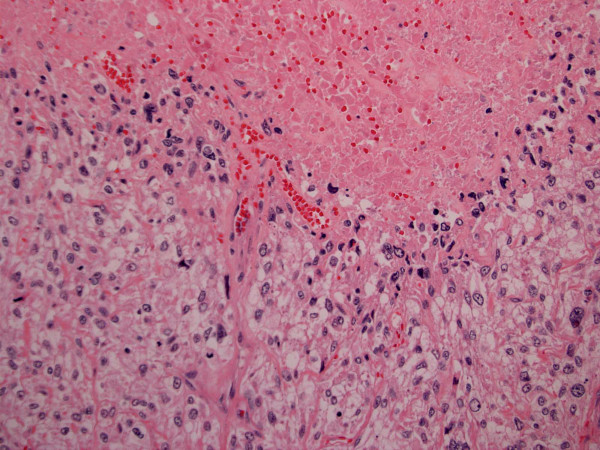
This pathologically malignant PEComa was also clinically malignant.

There have been three major attempts to correlate pathologic parameters of PEComas with patient outcome, in an effort to enumerate morphologic criteria predictive of malignant potential [5,8,13]. Folpe et al [13] derived their proposed criteria from a review of PEComas reported from a wide variety of anatomic locations. The resultant classification included 3 groups: 1) A "*Benign*" group in which none of 22 cases displayed aggressive behavior and which was characterized by tumor non-infiltrativeness, tumor size less than 5 cm, non high nuclear grade, lack of high cellularity, mitotic rate ≤ 1/50 high power fields (HPF), no necrosis and no vascular invasion; 2) A "*malignant*" group in which 12 (71%) of 17 cases displayed aggressive behavior and which was characterized by tumors grater than 5 cm, infiltrativeness, high nuclear grade and cellularity, necrosis, lymphovascular invasion and a mitotic rate ≥ 1/50 HPF; and 3)a group of tumors of "*uncertain malignant potential*" that were characterized by tumors with nuclear pleomorphism/multinucleated giant cells [0 (0%) of 6 displaying aggressive behavior] or size greater than 5 cm only [2 (12%) of 17 cases displaying aggressive behavior] [13]. In our own aforementioned analysis of 31 corpus cases which were classified based on patient outcome into "*Non-Malignant*" and "*Malignant*" groups, significant differences were found between these 2 groups regarding 3 pathologic parameters [8]. Regarding tumor size, the malignant cases (average size 9.6 cm) were significantly larger than their non-malignant counterparts (average size 4.67 cm, p = 0.04). However, there were no size thresholds that in of themselves could classify even 75% of the cases in both groups. The presence of coagulative necrosis (Figure 2) was highly associated with the malignant group [present in 9 (82%) of 11 cases], as compared with only 2 (11.8%) of the 17 cases in the non-malignant group (p = 0.0002). Finally, a mitotic count of ≤ 1 mitotic figures/10 HPF was found in 16 (88%) of the 18 non-malignant cases but in only 4 (40%) of the 10 malignant cases (p = 0.01). Although the possibility of suboptimal sampling remains, it is noteworthy that some uterine PEComas have been reported in which metastases developed in the absence of mitotic activity [23,29]. As such, lack of mitotic figures may not be necessarily reassuring for non-malignancy. Application of the Folpe et al [13] criteria to the uterine corpus cases showed that it classified 12 of the 13 malignant cases under our paradigm [8] appropriately into a malignant group. The 13^th ^case [32] would probably be classified as being of uncertain malignant potential. Because nuclear atypia and nuclear pleomorphism were inconsistently defined in many of the reported cases of uterine PEComa, The Folpe et al [13] criteria would probably also classify most of our "non-malignant" cases into their "uncertain malignant potential" group simply because of nuclear pleomorphism. Furthermore, "infiltrativeness", which presumably encompasses the "tongue-like" myometrial growth pattern in some uterine PEComas, would remove a tumor that otherwise qualifies from the "benign" group in the Folpe et al [13] classification, which is at best questionable regarding correlation with outcome. None of the 3 cases with this infiltrative pattern (and follow-up) in the series of Vang and Kempson [33], for example, recurred. The case associated with intraabdominal PEComatosis reported by Fadare et al [5] was otherwise histologically benign and also did not recur.

As with conventional smooth muscle neoplasms of the uterus, there are no morphologic criteria that can uniformly predict the malignant potential of all cases. Nonetheless, the presence of coagulative necrosis and/or a mitotic index >1 mitotic figure (MF)/10 HPF is highly worrisome for malignancy and patients with such tumors should be managed as such. However, a mitotic index >1 MF/10 HPF is typically seen in association with other features such as necrosis and cytologic atypia. Cases that can be classified as benign according to Folpe et al criteria [13] should be reported as being highly unlikely to display aggressive behavior based on limited evidence. In the author's opinion, until more cases are described and prognostic criteria become more finessed, all patients diagnosed with uterine PEComas should receive long-term follow-up irrespective of the pathologic designation because there is an inherent element of unpredictability to these neoplasms.

## Perivascular epithelioid cell tumors and some myomatous tumors of the uterus have overlapping clinicopathologic features

The principal controversial aspect of uterine PEComas stems from the fact that they have a distinct clinicomorphologic and immunophenotypic overlap with some smooth muscle neoplasms of the myometrium, which has called into question the validity of their segregation as a distinct clinicopathologic entity. In the simplest analysis of the overlap, smooth muscle neoplasia may be viewed as uterine tumors that *always *display myomatous differentiation and *occasionally *display melanocytic differentiation, whereas PEComas *always *display melanocytic differentiation and *usually *displays some myomatous differentiation. From this construct, it becomes apparent how controversies may arise regarding which end of this putative spectrum is best considered a variant of the other and which designation is most appropriate to apply for a given case that falls within the areas of overlap. These issues are discussed below.

### Clinicomorphologic overlap

As noted previously, uterine PEComas are defined morphologically by their constituent epithelioid and/or spindle cells with eosinophilic to clear cytoplasm. However, the uterine mesenchymal tumors that have traditionally been diagnosed as epithelioid leiomyomas/leiomyosarcomas (and all their historic appellations: leiomyoblastoma, clear cell leiomyoma/leiomyosarcoma, plexiform leiomyoma etc) are defined in a broadly similar fashion [54-61].

Epithelioid smooth muscle tumors of the uterus (ESM) may grow in nests, cords or diffuse sheets. Their constituent cells should be predominantly epithelioid and typically display eosinophilic cytoplasm, although a clear appearance may be seen in approximately 25% of cases [54-57]. They generally do not display the vascular network that is characteristic of PEComas. Stromal hyalinization is a potential feature of both tumors and may be extensive in both [8,55]. Both tumors may display multinucleated giant cells [8,13,55]. Extracellular myxoid material and osteoclast-like giant cells have been described in rare cases of ESM [55].

The author examined historical data on the prognostic clinicomorphologic features associated with those cases reported as ESM to determine whether these are broadly similar to those reported for uterine PEComas. First, cases reported as uterine PEComas and ESM occurred in patients in approximately the same age group (48 years for ESM [54] and 45 years for PEComa, see above]. Second, approximately 12–40% of cases reported as ESM pursued a malignant clinical course as compared with 43–44% of uterine PEComas (see above). However, the data on ESM is based on series of consecutive cases [54-57], whereas the data on PEComas is based on isolated case reports and small series, which probably artificially inflated the latter due to selection bias. Third, morphologic criteria are significantly less predictive of biologic behavior in ESM when compared with their conventional (i.e. non-epithelioid) counterparts [17], which may theoretically be an indicator of a tumor group that is too broadly defined. In their series of 26 ESM, Kurman and Norris [54] found significant overlap regarding morphologic features between malignant and benign cases. However, the presence of clear cells, stromal hyalinization, an expansile tumoral margin and the absence of necrosis were associated with benign behavior, whereas tumor size 6 cm or larger, extensive necrosis, infiltrating margins and mitotic activity of ≥ 5 MF/10 HPF were more associated with malignant behavior. The authors proposed the then provisional criteria of ≥ 5 MF/10 HPF as the threshold that defines malignancy. The Kempson group [57,61] found that all cases with tumor necrosis behaved in a malignant fashion, but that the absence of necrosis and cytologic atypia did not guarantee a benign course if there is more than 5 MF/10 HPF. Seven cases in which there was no tumor necrosis, no more than "minimal" atypia and <5 MF/10 HPF pursued a benign course. The authors recommended that tumors with <5 MF/10 HPF, no necrosis, and moderate to severe atypia be designated as being of "uncertain malignant potential". Oliva et al [56] confirmed the importance of mitotic activity: for uterus-confined cases, a mitotic index of 2–4 MF/10 HPF was malignant in approximately 50%. Prayson et al [55] did not find any single morphologic criteria predictive of malignancy, but the authors noted that their clinically malignant cases typically displayed tumor necrosis, cytologic atypia and mitotic activity of at least 3–4 MF/10 HPF. All of these criteria are applicable to cases reported as PEComas. Any uterine PEComa with coagulative necrosis and/or a mitotic index >5 MF/10 HPF will be classified as malignant by either of the paradigms outlined above [8,13]. The mitotic index threshold that increases the probability of aggressive behavior seems to be much lower for uterine PEComas, however, the author is unaware of any uterine PEComas in which increased mitotic activity is the only worrisome feature (i.e. a tumor with greater than 1 MF/10 HPF unassociated with tumor necrosis and/or cytologic atypia). Furthermore, as noted previously, at least 2 uterine PEComas have pursued a malignant course in the absence of any mitotic activity, although the level of sampling in those cases is unclear [23,29].

Undoubtedly, some morphologic overlap exists between cases reported as PEComas and those reported as ESM. To summarize the above comparative data regarding clinicomorphologic features, both tumors may display clear cells, epithelioid cells, stromal hyalinization and multinucleated giant cells. A delicate vascular network characterizes PEComas but not ESM. Both are seen in patients in the same age group. Both tumors can probably be classified as malignant if there is coagulative necrosis and/or >5 MF/10 HPF. The implications of mitotic activity below the latter threshold seems to differ between both neoplasms, although the data is limited.

### Immunophenotypic overlap

Uterine PEComas definitionally display at least focal immunoreactivity for melanocytic markers [8,10-15]. However, it appears that a substantial subset of uterine smooth muscle tumors can also be positive for melanocytic markers [58,59,62-67]. Three studies [62-64], although published only in abstract form, found at least focal HMB-45 immunopositivity in 54 (65%) of 83 leiomyomas. Furthermore, at least focal imunoreactivity for melanocytic markers other than HMB-45 [Melan A and miTF] were reported in 2 (22%) of 9 and 5 (100%) of 5 leiomyomas [64]. In contrast, the 16 leiomyomas tested by Bosincu et al [24] were all HMB-45 negative. HMB-45 positivity has also been demonstrated in leiomyosarcomas [58,59,64-66]. Oliva et al [64] reported HM45 positivity in 21 (31%) of 67 leiomyosarcomas, with most cases co-expressing Melan A. Simpson and Albores-Saavedra [65] recently confirmed those findings, reporting at least focal HMB-45 positivity in 36% of conventional leiomyosarcomas. HMB-45 positivity was more frequently found in poorly differentiated leiomyosarcomas as compared with their well-differentiated counterparts [65]. Silva et al [58] found that HMB-45 positivity in ESM is generally localized to the clear cell areas. The authors selected 5 leiomyosarcomas with clear cell areas and at least focal desmin/caldesmon positive spindle areas from a group of 12 epithelioid leiomyosarcomas. Four (80%) of the 5 cases were found to be HMB-45 positive only in the clear cell areas. The 5^th ^case had less than 1% clear cells and was HMB-45 negative [58]. The latter findings were confirmed by Hurrell and McCluggage [66] who found that no immunoreactivity for HMB-45 in 3 epithelioid leiomyosarcomas without clear cell areas but clear cell-localized HMB-45 positivity in 2 other epithelioid leiomyosarcomas with clear cell areas. Five (56%) of 9 ESM were found to be HMB-45 positive in another study, although the extent of cytoplasmic clarity in those cases was not stated [64].

Because the normal myometrium may also be HMB-45 positive [62,63], and because the immunoreactivity has been demonstrated for multiple melanocytic markers (miTF, HMB-45 and Melan A), it is unlikely that this reaction is merely artifactual. Melanocytic differentiation appears to be an intrinsic biologic property of a subset of smooth muscle tumors. It is noteworthy that HMB-45 positivity was only demonstrable in the metastatic deposit of one case of uterine leiomyosarcoma [59], and that HMB-45 positivity was associated with poor tumor differentiation in a series of others [65]. As the author has noted previously [6,8], there are established links between melanocytic and smooth muscular differentiation in other contexts. Among these is the expression of smooth muscle actin in some cutaneous melanomas [68] and pigmentation in some myomatous tumors of the uterus [69]. Furthermore, cells derived from neural crest, the origin of melanocytes, have been shown in animal model embryologic studies to give rise to perivascular as well as branchial smooth muscle cells [70,71]. Finally, *in vitro *studies have also shown that neural crest cells have the potential for differentiating into smooth muscle cells in the presence of folic acid [72], transforming growth factor-beta [73] and some specific media [74].

To summarize the comparative data, approximately one-third of conventional leiomyosarcomas may display at least focal immunoreactivity for melanocytic markers, and this should not alter their designation as leiomyosarcomas. ESM may also be positive for HMB-45, predominantly in the clear cell areas, but probably also in the cells with cytoplasmic eosinophilia. Keratin positivity may be found in both ESM and uterine PEComas, but is significantly more frequently positive in former than the latter [8,75]. Desmin positivity is present in approximately 50% of both ESM [76] and PEComas [8]. In analyses of a total of 7 epithelioid leiomyosarcomas with clear cell areas, smooth muscle actin was found to be diffusely positive in 6 and focally positive in 1 [58,66]. Smooth muscle actin is positive in 80% of PEComas in general [13] and in 73% of uterine PEComas [8]. As such, cases reported as ESM and uterine PEComa display a substantial immunophenotypic overlap which, at minimum, is indicative of their shared lines of differentiation. The few cases of ESM that have been examined ultrastructurally have shown their constituent cells to be comprised of numerous vacuolated mitochondria [77], cytolysosomes and glycogen aggregates [78]. Melanosomes were identified in one uterine PEComa [31], but not in another [30].

## Uterine PEComas and epithelioid smooth muscle tumors: a practical approach

Given the aforementioned extent of clinicomorphologic and immunophenotypic overlap that exists between uterine PEComas and ESM (Table 3), and since 42 (95%) of the 44 uterine PEComas were reported since the beginning of 2000, the logical question arises as to whether PEComas are a distinct clinicopathologic entity or whether they merely represent a selected group of HMB-45 positive smooth muscle tumors. In the author's opinion, this is a false choice. First, the discussion has to be "purified" by the stipulation that a uterine mesenchymal tumor should not be designated a PEComa simply because it is positive for HMB-45 or some other melanocytic marker. There is no significant debate at this junction that conventional smooth muscle tumors (i.e. tumors that are comprised predominantly of fascicles of spindle cells with eosinophilic cytoplasm) can display immunoreactivity for melanocytic markers [64,65]. When this group of tumors is removed the discussion can then be centered on uterine mesenchymal tumors that are comprised predominantly of epithelioid cells or an admixture of epithelioid and spindle cells throughout the tumor.

**Table 3:** A comparison of the clinicopathologic features of uterine PEComas and uterine epithelioid smooth muscle tumors.

**Feature**	**PEComa**	**Epithelioid smooth muscle tumors**	**References**
Average patient age	45 years	48 years	54,◙
Epithelioid cells	+	+	8,54,55
Spindle cells	+	+	8,54,55
Multinucleated giant cells	+	+	5,8,13,41,55
Stromal Hyalinization	+	+	8,19,25,33,35, 39,41,54,55
Network of capillaries	+	-	8,13,54,55
Clear cells	Usual	Occasional	8,10–15, 54,55,58,66
Eosinophilic cells	Usual	Usual	
Immunopositivity for at least one melanocytic marker	100%	Up to 56%	8,10–15,64
Desmin immunopositivity	49%	50%	8,76
Smooth muscle actin immunopositivity	73%	Up to 100%	8,58,66
Proportion of reported cases associated with TSC	9.1%	No published data	◙

◙see text; TSC tuberous sclerosis complex

A clinicopathologic entity should be pathologically (via morphologic evaluation and/or ancillary techniques) definable and have clinical significance. ESM have been so classified for at least three decades. However, their behavior has been notoriously difficult to predict from morphologic criteria, which may be considered as evidence of excessive heterogeneity in tumors currently defined as ESM and the need to delineate biologically relevant subsets within them. PEComas and ESM have significant similarities but also significant differences (Table 3). Indeed, an argument can be advanced that it is no more invalid a position to consider PEComas as a variant of ESM than it is to consider all previously reported ESM as variants of PEComas. What is undisputable is that these tumors share lines of differentiation. It is an impediment to scientific progress when scientists take rigid, dogmatic positions simply because of tradition. One approach to resolving the PEComa versus ESM question is to postulate that a given tumor is better characterized into a diagnostic category if pathologically similar, extrauterine tumors are well described. In this respect, it is noteworthy that epithelioid smooth muscle tumors are decidedly rare outside of the uterus. Most of the extrauterine (predominantly retroperitoneal and gastrointestinal) tumors that were previously diagnosed as epithelioid smooth muscle tumors are now considered epithelioid gastrointestinal stromal tumors. In contrast, PEComas are considered anatomically ubiquitous. One comparative genomic hybridization study found a closer kinship, regarding the patterns of chromosome losses and gains, between a uterine PEComa and extrauterine PEComas than between the uterine PEComa and uterine smooth muscle neoplasia [53]. In animal models carrying a germline mutation to the tuberous sclerosis 2 (TSC2) gene, there is an increased predisposition to develop uterine and extrauterine tumors [79]. Notably, a disproportionate percentage of the uterine tumors, which the authors classified as leiomyomas/leiomyosarcomas based on morphology and desmin/actin immunoreactivity, were of the epithelioid type. PEComas, of course, have a known association with TSC2 gene alterations [80]. Second, a recent study reported a 100% rate of CD1a immunoexpression in PEComas from various sites [81]. The author examined 5 ESM (4 epithelioid leiomyomas and 1 epithelioid leiomyosarcoma) diagnosed by WHO criteria [17] and all were negative for CD1a. (Fadare O, unpublished data, 2008). Other selectively noteworthy findings in uterine PEComas include their 9.1% association with TSC, their occasional occurrence in young patients [23,29,34], and their occasional display of metastases in the absence of significant tumor mitotic activity [23,29]. Although the precise etiopathogenesis of PEComas remain to be elucidated, the findings outlined above argue for PEComas as a distinct entity or at least a specific subset of smooth muscle neoplasia. The recent demonstration of elevated phospho-p70S6K and reduced phospho-AKT expression, indicative of mTOR (mammalian target of rapamycin) activation, in a group of extra-renal PEComas raises the possibility of using mTOR inhibitors such as rapamycin in the treatment of uterine PEComas and is another argument for their routine segregation [82].

The author shares the opinion that has been expressed by others that most PEComas can be morphologically distinguished from classical epithelioid smooth muscle tumors by their distinctive network of capillaries [13]. Nonetheless, these lesions may exist at different points on a single clinicomorphologic spectrum [33], and their distinction may admittedly be difficult. Future studies should evaluate a series of archival epithelioid smooth muscle tumors to determine whether cases that are morphologically and immunophenotypically more consistent with PEComas are identifiable, and perhaps more importantly, whether these cases are prognostically distinct from the remaining cases in the group. At present time, the author proposes this practical approach to these neoplasms is as follows:

1) Predominantly conventional smooth muscle neoplasms that display immunoreactivity for one or more melanocytic markers should be diagnosed as leiomyosarcomas/leiomyomas, with a comment about their melanocytic differention. This is designed to segregate these cases because the significance of melanocytic differentiation is unknown, and melanocytic differentiation may emerge at a metastatic site [59], with the attendant potential for the misdiagnosis of the metastatic lesion as a primarily melanocytic malignancy. The presence of clear or epithelioid cells in an otherwise predominantly conventional smooth muscle neoplasm should not affect its designation as such.

2) All epithelioid mesenchymal neoplasms of the uterus should be tested for melanocytic markers, to include at least HMB-45.

3) Tumors with *absolutely *characteristic morphologic and immunophenotypic features are diagnosed as a perivascular epithelioid cell tumor (PEComa). However, given the current state of evidence, it is no longer advisable to use "PEComa" as a diagnostic term in isolation, as it is akin to diagnosing a uterine mesenchymal tumor as a "smooth muscle tumor" without further description. Rather, there should be some statement regarding their expected biologic behavior as predicted by morphologic characteristics. PEComas should be described as being highly likely to display aggressive behavior if they display coagulative necrosis. A mitotic index of >1/10 HPF is highly worrisome for malignancy but have been uniformly seen concurrent with other features such as cytologic atypia and necrosis. PEComa cases that can be classified as benign according to Folpe et al criteria [13] should be reported as being highly unlikely to display aggressive behavior based on limited evidence. All other cases (PEComas) are of uncertain malignant potential. It is recommended that all patients diagnosed with uterine PEComas receive long-term follow-up irrespective of the pathologic designation due to the unpredictability to these neoplasms.

4) Epithelioid mesenchymal tumors that do not display all the characteristic features of PEComa should be diagnosed as epithelioid leiomyomas, epithelioid leiomyosarcomas or epithelioid tumors of uncertain malignant potential (if they otherwise have the features of these lesions) per aforementioned criteria [17,54-57,61]. As with their conventional counterparts, melanocytic differentiation should similarly be noted if present.

5) Finally, as with all neoplasms, adequate sampling is of paramount importance to ensure that all areas of these uterine mesenchymal tumors are being optimally represented microscopically.

## Competing interests

The author(s) declare that they have no competing interests.

## Authors' contributions

OF wrote the manuscript.
